# Morphologies and composition changes in nonculprit subclinical atherosclerosis in diabetic versus nondiabetic patients with acute coronary syndrome who underwent long-term statin therapy

**DOI:** 10.1038/s41598-023-32638-w

**Published:** 2023-04-01

**Authors:** Pei-na Meng, Jia-cong Nong, Yi Xu, Wei You, Tian Xu, Xiang-qi Wu, Zhi-ming Wu, Bi-lin Tao, Ya-jie Guo, De-lu Yin, Hai-bo Jia, Song Yang, Fei Ye

**Affiliations:** 1 Department of Cardiology, Nanjing First Hospital, Nanjing Medical University, 68 Changle Road, Nanjing, 210006 China; 2 Department of Epidemiology and Biostatistics, Center for Global Health, School of Public Health, Nanjing Medical University, 101 Longmian Ave., Nanjing, 211166 China; 3 Department of Cardiology, The First Hospital of Lianyungang, Xuzhou Medical University, No. 6 East Zhenhua Road, Haizhou District, Lianyungang, 222061 China; 4 Department of Cardiology, The People’s Hospital of Yixing City, 75 Tongzhenguan Road, Yixing, 214200 China

**Keywords:** Health care, Medical research

## Abstract

Although patients are undergoing similar lipid-lowering therapy (LLT) with statins, the outcomes of coronary plaque in diabetic mellitus (DM) and non-DM patients are different. Clinical data of 239 patients in this observational study with acute coronary syndrome was from our previous randomized trial were analyzed at 3 years, and 114 of them underwent OCT detection at baseline and the 1-year follow-up were re-anlayzed by a novel artificial intelligence imaging software for nonculprit subclinical atherosclerosis (nCSA). Normalized total atheroma volume changes (ΔTAVn) of nCSA were the primary endpoint. Plaque progression (PP) was defined as any increase in ΔTAVn. DM patients showed more PP in nCSA (ΔTAVn; 7.41 (− 2.82, 11.85) mm^3^ vs. − 1.12 (− 10.67, 9.15) mm^3^, *p* = 0.009) with similar reduction of low-density lipoprotein cholesterol (LDL-C) from baseline to 1-year. The main reason is that the lipid component in nCSA increases in DM patients and non-significantly decreases in non-DM patients, which leads to a significantly higher lipid TAVn (24.26 (15.05, 40.12) mm^3^ vs. 16.03 (6.98, 26.54) mm^3^, *p* = 0.004) in the DM group than in the non-DM group at the 1-year follow-up. DM was an independent predictor of PP in multivariate logistic regression analysis (OR = 2.731, 95% CI 1.160–6.428, *p* = 0.021). Major adverse cardiac events (MACEs) related to nCSA at 3 years were higher in the DM group than in the non-DM group (9.5% vs. 1.7%, *p* = 0.027). Despite a comparable reduction in LDL-C levels after LLT, more PP with an increase in the lipid component of nCSA and a higher incidence of MACEs at the 3-year follow-up was observed in DM patients.

*Trial registration*: ClinicalTrials.gov. identifier: NCT02140801.

## Introduction

Even with the widespread use of lipid-lowering therapy (LLT)^1–5^ and progress in the study of atherosclerotic mechanisms^5–9^, cardiovascular events related to atherosclerosis remain the leading cause of mortality and morbidity worldwide^10,11^. Because atherosclerosis is a multifactorial disease, the pure LLT effect on coronary atherosclerosis is weakened in patients with multiple risk factors, such as diabetes mellitus (DM), compared with others despite the comparable reduction in low-density lipoprotein cholesterol (LDL-C)^3,12^. Therefore, the current concept of atherosclerosis treatment should not be limited to the extent and duration of LDL-C reduction and should also focus on the outcome (progression or regression) of plaque after comprehensive treatment, which is more related to the corresponding incidence of related cardiovascular events^3,8,9,13^.

With the development of intravascular imaging (IVI) technology, quantitative and qualitative studies of coronary atherosclerosis have increasingly confirmed the importance of LLT for plaque regression, but the results of DM seem to be unsatisfactory^14,15^. Disorders of glucose metabolism may be the main factors leading to the progression and formation of coronary plaque and the reduced effect of LLT^6,16^. Plaque stabilization and regression are clinically desirable outcomes, which can be reflected by changes in normalized total atheroma volume (ΔTAVn) or percent atheroma volume (ΔPAV) and plaque composition in luminal imaging^9,17–19^. Although previous studies on plaque components were mainly based on quantitative analysis of the virtual histology of intravascular ultrasound (IVUS) and showed that it was correlated with clinical outcomes^20–22^, the correlation between plaque components and histopathology still needs to be further explored^23,24^. More studies are based on semiquantitative analysis of optical coherence tomography (OCT) data, especially for lipids, calcified plaque and fibrous cap thickness, but the quantification of plaque components based on OCT is insufficient^9,25,26^. Recently, an artificial intelligence (AI) framework named the optical flow ratio (OFR) based on OCT data was used to automatically analyse the components and volume of coronary plaque precisely for clinical use (Pulse Medical Imaging Technology, Shanghai, Co., Ltd.)^18,27,28^. The purpose of this post hoc analysis was to compare changes in plaque morphology and composition in nonculprit subclinical atherosclerosis (nCSA) by analysing the OFR in DM patients versus patients without DM (non-DM) at the 1-year follow-up. Further differences in major adverse cardiac events (MACEs) related to nCSA at the 3-year clinical follow-up were also compared.

## Methods

### Study population and design

**Figure 1:**
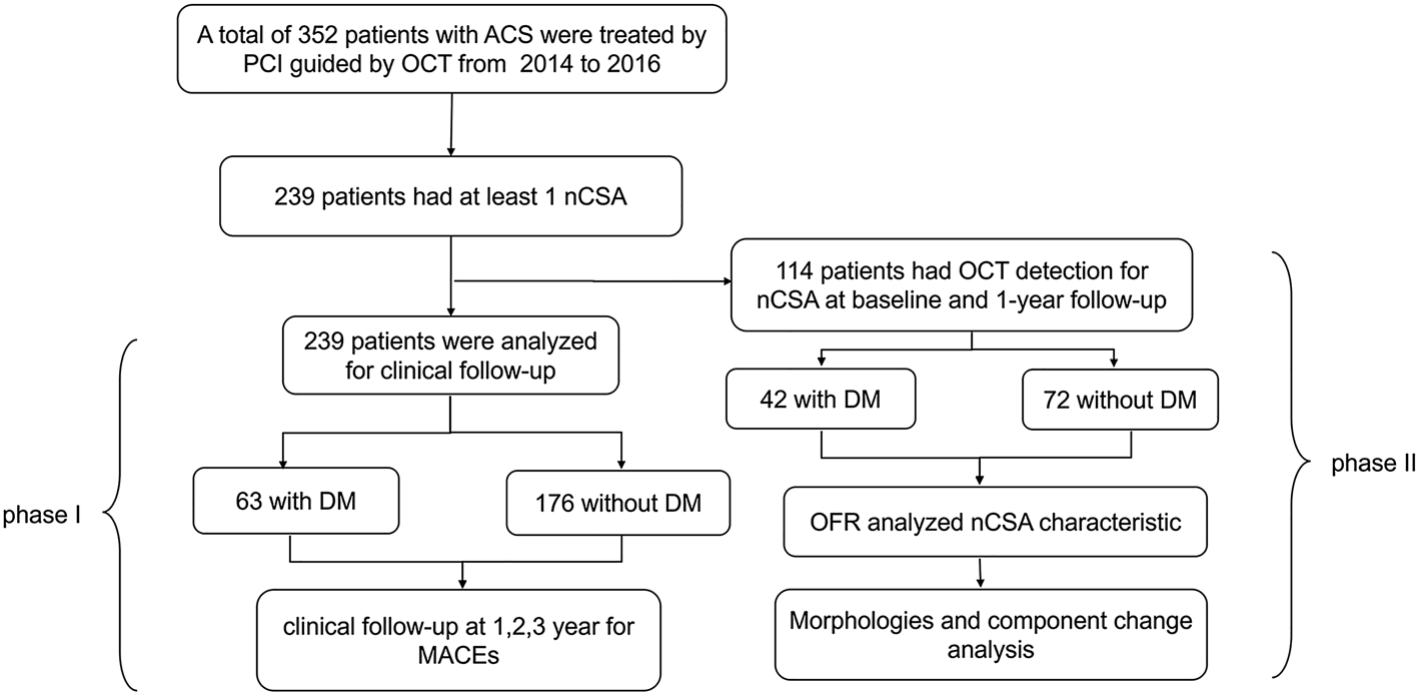
Study flow chart.

All the eligible patient data were taken from our previous randomized study to assess dynamic natural morphologies and component changes using OFR analysis in nCSA (de novo lesions, which were defined as those containing 30–70% diameter stenosis by visual estimation on angiography without any percutaneous coronary intervention (PCI) treatment of patients who underwent routine long-term LLT with statins (ClinicalTrials.gov. Number: NCT02140801)^29^. Based on the criteria of the American Diabetic Association for type 2 DM^30,31^, a total of 239 acute coronary syndrome (ACS) patients, with 63 DM cases (26.4%) and 176 non-DM cases (73.6%), who underwent PCI for culprit lesions and at least one nCSA were enrolled in the phase I analysis with a clinical follow-up of at least 3 years for MACEs (defined as a composite of cardiac death, myocardial infarction, and ischaemia-driven revascularization) related to nCSA information collection. Among them, 114 patients who underwent OCT detection at baseline and 1-year follow-up for nCSA entered into phase II analysis for morphologies and composition of nCSA measurement by OFR, which included 42 DM cases (36.8%) and 72 non-DM cases (63.2%) (Fig. 1). The exclusion criteria were type 1 DM, history of PCI or coronary artery bypass grafting before, absence of nCSA or nCSA combined with thrombosis/aneurysm/ectasia, incomplete OCT data or low-quality OCT data, which prevented OFR analysis, and lack of clinical follow-up data. If a nCSA lesion was located in a PCI-treated coronary artery, the distance between the stent edge and the analysed location was at least 10 mm or more. In all cases, only one nCSA with plaque burden > 30% and < 70% was examined per patient. Some cases might have multiple nCSA, especially in the DM group, we firstly selected lesions with high OCT image quality, heavy plaque burden, complete OCT follow-up data, and which located at proximal part of coronary artery as target nCSA for analysis. If multiple similar nCSA still existed, a target nCSA was selected in the order of left anterior descending (LAD), right coronary artery (RCA), and left circumflex (LCx). All patients signed an informed consent form, and the study was approved by the ethics committee of Nanjing First Hospital. All the methods performed in our study were in accordance with the relevant guidelines and regulations.

### Medical therapy and clinical follow-up

All patients received dual antiplatelet therapy, such as aspirin 100 mg/d and clopidogrel 75 mg/d or ticagrelor 90 mg bid, for at least 1 year followed by single antiplatelet therapy with aspirin 100 mg/d routinely nested. Statin-based LLT (rosuvastatin 10 mg/d or atorvastatin 20 mg/d) was routinely used in all patients post PCI. Ezetimibe could be added if the doctor considered it necessary, but no patients received proprotein convertase subtilisin/kexin type 9 inhibitors (PCSK9i) because such medication was not available in China at that time. Other risk factor (such as hypertension and glycaemic control in patients with DM) treatments were performed at the doctor’s discretion. Lipid profile data, such as total cholesterol, LDL-C, high-density lipoprotein cholesterol (HDL-C) and triglyceride, at baseline and the 1-year follow-up were collected and registered by the staff of the follow-up group in the cardiology department of Nanjing First Hospital. Changes in lipid profiles were calculated as follows: changes in total cholesterol (ΔTotal cholesterol) = total cholesterol value at the 1-year follow-up minus total cholesterol value at baseline; changes in LDL-C (ΔLDL-C) = LDL-C level at the 1-year follow-up minus LDL-C level at baseline; and so on, similar calculations were performed for changes in HDL-C (ΔHDL-C), changes in non-HDL-C (Δnon-HDL-C), and changes in triglycerides (Δtriglycerides). Percent changes in LDL-C (ΔLDL-C%) were calculated as ΔLDL-C/LDL-C level at baseline × 100. At the same time, clinical follow-up data at 1, 3, 6, 12, 24 and 36 months post PCI, such as the occurrence of death, myocardial infarction, recurrent angina, or revascularization events, were obtained through outpatient or telephone follow-up, and finally, the clinical event committee determined whether the event was related to nCSA.

### OCT image acquisition and preliminary analysis

Both ILUMINE OPTIS and C7-XR (Lightlab Imaging Incorporated, Westford, MA) could be applied by a 2.7F (Dragonfly OPTIS or Dragonfly Duo imaging catheter, Westford, MA) catheter automatic pullback system at a speed of 36 mm/s with continuous contrast injection for red blood cell removal. To obtain high-quality images of the coronary artery wall, plaque characteristics and avoiding coronary spasm, OCT images were acquired after nitroglycerin intracoronary injection, and each procedure was in strict accordance with the consensus recommendations^32^. If there were no influence of red blood cell and OCT artefacts for OCT images, the range of OCT detection included the target vessel segment at baseline and 1-year follow-up. All OCT images were submitted for offline analysis to the MGH OCT core laboratory, where the images were analysed by two independent investigators who were blinded to the clinical data using an offline review workstation (Offline software E.5 OPTSTM System, Abbott) to qualitatively analyse plaque properties and semiquantitatively analyse microchannels intra plaques.

### Quantitative analysis of OFR for plaque characteristics

All OCT data were saved in DICOM format and analysed offline by OFR software (OctPlus, version V2, Pulse Medical, Shanghai, China) with AI algorithms^27,28^. Quantitative planimetry measurements were acquired by automatic border detection for the adventitia and inner margin of the lumen by manual correction frame by frame. After manual multiaspect coregistration based on reproducible index side branches, known pullback speeds (which could be measured by the quantified frame number with an interval of 0.2 mm per frame) and combined with angiographic results to ensure that study segments analysed by OFR at baseline and 1-year follow-up were consistent. The segment of interest for OFR measurement was defined as at least 15 mm long of maximal plaque-containing region (at least plaque burden of ≥ 30%). The measurement index is summarized as follows as in previous studies^18,33^: total atheroma volume (TAV), percent atheroma volume (PAV), fibrous TAV or PAV, calcium TAV or PAV, macrophage TAV or PAV, and thinnest fibrous cap thickness (TFCT) in the analysed segment. The normalized TAV (TAVn) was calculated as TAVn = TAV/number of frames of measurement segment × 100 to balance the difference between the absolute value of the lesion in different patients. Changes in TAV (ΔTAV) were defined as the TAVn at the 1-year follow-up minus the TAVn at baseline, and changes in PAV (ΔPAV) had a similar definition as follows: ΔPAV = PAV at the 1-year follow-up—PAV at baseline; in a similar calculation, changes in fibrous TAV or PAV (fibrous ΔTAV or ΔPAV) = fibrous TAV or PAV at the 1-year follow-up—fibrous TAV or PAV at baseline; changes in lipid TAV or PAV (lipid ΔTAV or ΔPAV) = lipid TAV or PAV at the 1-year follow-up—lipid TAV or PAV at baseline; changes in macrophage TAV or PAV (macrophage ΔTAV or ΔPAV) = macrophage TAV or PAV at the 1-year follow-up—macrophage TAV or PAV at baseline; changes in TFCT (ΔTFCT) = TFCT at the 1-year follow-up—TFCT at baseline. Meanwhile, the minimal luminal area (MLA) in the analysed segment was measured directly by OFR software and compared with the same point between baseline and 1-year follow-up by coregistration. Changes in MLA (ΔMLA) were defined as MLA at the 1-year follow-up—MLA at baseline at the same point. Plaque regression (PR) was defined as any decrease in ΔTAVn; otherwise, plaque progression (PP) was defined as any increase in ΔTAVn^34,35^. Microchannels were defined as nonsignal tubular structures without three layers of arterial wall (i.e., intima, media, and adventitia) in cross-sectional and longitudinal profiles^32,36,37^ and were classified into two types depending on the distal location according to a previous study as follows: external running type was defined as located in the adventitia, and internal running type was defined as located within plaque^38^.

### Consistency test of OCT and OFR measurement

All OCT data analyses were performed by two independent technicians (trained and certified by Pulse Medical Imaging Technology, Shanghai, China, Co., Ltd., before stating measurements) without knowledge of the patient's clinical situation. Intraobserver and interobserver variability of the OCT image analysis by OFR were assessed for fifty randomly selected data for plaque characteristics qualitative analysis by Kappa statistics (for categorical variables: lipid, fibrous, and calcium characteristics) or intraclass correlation coefficients (ICCs, for continuous variables: TAV, PAV, and TFCT) by the same technician at a 2-week interval or by 2 independent technicians. Finally, all kappa > 0.9 and all ICC > 0.9 were obtained by consistency tests.

### Study endpoints

The primary endpoint was the difference in ΔTAVn/ΔPAV between DM and non-DM patients in the phase II analysis. The second endpoints included the difference in changes in plaque composition (such as lipid, fibrous, calcium and macrophage ΔTAVn/ΔPAV) of nCSA between DM and non-DM patients in the phase II analysis. The other exploratory endpoint was the difference in the incidence of MACEs related to nCSA at the 3-year clinical follow-up between the two groups in the total population of our previous randomized study (phase I analysis).

### Statistical analysis

All continuous variables were described as the mean ± standard deviation or median (interquartile range), and differences between groups were analysed using Student’s *t* test or the nonparametric test. Categorized variables were described in terms of percentages and analysed using the chi-square test. Univariate correlation analysis was performed firstly, and if *p* < 0.1, multivariate logistic regression analysis was performed based on stepwise regression method according to minimum Akaike information criterion. The logistic regression model was used to estimate the association between PP/PR and clinical features (including age, sex, DM/non-DM, medical therapy project, and lipid profile). The odds ratio (OR) and 95% confidence interval (CI) were used to estimate the strength of the relationship. MACEs-free survival curves of patients with or without DM were produced using the Kaplan‒Meier method and were compared by the log-rank test.

All statistical tests were two-tailed, and the significance level was set at 0.05. All analyses were performed using R software for Windows version 4.1.2 (https://www.r-project.org/).

### Ethics approval

The study was approved by the ethics committee of Nanjing First Hospital.

### Consent to participate

All patients signed informed consent to participate in the study.

## Results

### Clinical general data

Baseline clinical characteristics, including traditional risk factors correlated with coronary artery disease, lipid profile, glucose, parameters of renal function, location of nCSA and medical therapy regimen, of patients with or without DM are summarized in Table 1 (total population in the phase I analysis) and Table 2 (OCT detection population in the phase II analysis). There were no significant differences in clinical risk factors or lipid profiles at baseline or at the 1-year follow-up between the two groups; however, fasting glucose levels were increased (7.63 ± 2.97 mmol/L vs. 4.85 ± 0.89 mmol/L, *p* < 0.001) and total cholesterol levels were decreased (3.71 ± 0.85 mmol/L vs. 4.08 ± 1.02 mmol/L, *p* = 0.021) in the phase I analysis, and fasting glucose levels (7.74 ± 3.00 mmol/L vs. 5.03 ± 0.90 mmol/L, *p* < 0.001) in the phase II analysis were higher in the DM group than in the non-DM group at baseline. Furthermore, higher fasting glucose levels (7.59 ± 2.13 mmol/L vs. 5.45 ± 0.85 mmol/L, *p* < 0.001) and more reduction in Δtriglycerides (− 0.41 (− 0.90, − 0.02) vs. − 0.20 (− 0.56, 0.19), *p* = 0.037) in the phase I analysis were observed, and fasting glucose levels (7.56 ± 2.13 mmol/L vs. 5.45 ± 0.85 mmol/L, *p* < 0.001) in the phase II analysis were higher in the DM group than in the non-DM group at the 1-year follow-up. The changes in the lipid profile of total cholesterol, LDL-C, non-HDL-C and percent changes in LDL-C were not significantly different between the two groups, but the ratio of target achievement according to current guidelines (< 1.4 mmol/L) for patients with atherosclerotic cardiovascular disease was very low (20.5% vs. 20.5%, *p* = 0.310 in the phase I analysis, and 23.8% vs. 27.8%, *p* = 0.428 in the phase II analysis) in the DM group compared with the non-DM group^39–42^. All the patients received long-term LLT with statins, and 28% (67/239) of the patients received ezetimibe because LLT with statins alone was not as effective as doctors’ targets.

**Table 1:** Patient demographics in phase I analysis.

	Total (N = 239)	DM group (n = 63)	Non-DM group (n = 176)	*p* Value
Age, years	65.00 [58.00, 71.00]	66.00 [59.00, 74.00]	65.00 [58.25, 70.00]	0.191
Men n (%)	174 (72.8)	46 (73.0)	128 (72.7)	0.965
Body mass index	24.79 ± 3.13	25.39 ± 3.21	24.22 ± 3.09	0.120
Hypertension, n (%)	169 (70.7)	46(73.0)	123 (69.9)	0.640
Dyslipidemia, n (%)	170 (71.1)	49(77.8)	121 (68.8)	0.175
Smoking, n (%)	89 (37.2)	19 (30.1)	70 (39.8)	0.176
Baseline medical therapy				
Statin, n (%)	239 (100.0)	63 (100.0)	176 (100.0)	NA
Ezetimibe, n (%)	67 (28.0)	19(30.2)	48 (27.3)	0.662
Antiplatelet therapy, n (%)	239 (100.0)	63 (100.0)	176 (100.0)	NA
Clopidogrel, n (%)	146 (61.1)	31(49.2)	115(65.3)	0.024
Ticagrelor, n (%)	93 (38.9)	32(50.8)	61(34.7)	0.024
ACEI/ARB, n (%)	143 (59.83)	39 (61.9)	104 (59.1)	0.696
CCB, n (%)	52 (21.8)	14 (22.2)	38 (21.6)	0.917
Beta-blocker, n (%)	130 (54.4)	37 (58.7)	93 (52.8)	0.421
Baseline lipid profile				
Total cholesterol, mmol/L	3.98 ± 0.99	3.71 ± 0.85	4.08 ± 1.02	0.021
LDL-C, mmol/L	2.42 ± 0.84	2.24 ± 0.73	2.42 ± 0.86	0.079
HDL-C, mmol/L	1.02 ± 0.26	0.97 ± 0.22	1.05 ± 0.27	0.107
Non-HDL-C, mmol/L	2.95 ± 0.96	2.75 ± 0.84	2.97 ± 0.98	0.083
Triglycerides, mmol/L	1.95 ± 0.65	1.81 ± 0.95	2.00 ± 0.69	0.186
Glucose, mmol/L	5.78 ± 1.79	7.63 ± 2.97	4.85 ± 0.89	< 0.001
Serum creatinine	–	74.67 ± 20.4	75.14 ± 16.88	0.857
eGFR, ml	–	96.97 ± 26.97	93.94 ± 20.71	0.361
60 ml ≤ eGFR < 90 ml, n (%)	–	23(36.5)	71(40.3)	0.593
Location of nCSA				
LAD, n (%)	135(56.5)	42(66.7%)	93(52.8%)	0.163
LCx, n (%)	48(20.1)	10(15.8%)	38(21.6%)	
RCA, n (%)	56(23.4)	11(17.5%)	45(25.6%)	
1-year follow up of lipid profile				
Total cholesterol, mmol/L	3.62 ± 0.86	3.61 ± 0.80	3.61 ± 0.88	0.944
LDL-C, mmol/L	1.97 ± 0.72	1.94 ± 0.69	1.95 ± 0.74	0.925
LDL-C < 1.4 mmol/L, n(%)	57(23.8)	13(20.5)	44(25.0)	0.310
HDL-C, mmol/L	1.20 ± 0.29	1.17 ± 0.27	1.21 ± 0.29	0.348
Non-HDL-C, mmol/L	2.42 ± 0.83	2.45 ± 0.78	2.42 ± 0.85	0.821
Triglycerides, mmol/L	1.36 ± 0.65	1.36 ± 0.57	1.36 ± 0.69	0.983
Glucose, mmol/L	5.97 ± 1.52	7.59 ± 2.13	5.45 ± 0.85	< 0.001
Change in lipid profile between index and 1-year follow-up				
ΔTotal cholesterol, mmol/L	− 0.26 (− 0.98, 0.27)	− 0.19 (− 0.86, 0.62)	− 0.31 (− 1.05, 0.18)	0.129
ΔLDL-C, mmol/L	− 0.41 (− 1.08, 0.13)	− 0.28 (− 1.02, 0.23)	− 0.46 (− 1.10, 0.12)	0.350
ΔLDL-C%	− 19.94 (− 39.06, 6.41)	− 13.89 (− 39.11, 11.94)	− 21.07 (− 39.59, 5.31)	0.370
Δnon-HDL-C, mmol/L	− 0.46 (− 1.16, 0.18)	− 0.44 (− 1.06, 0.35)	− 0.46 (− 1.29, 0.13)	0.242
ΔHDL-C, mmol/L	0.19(0.02, 0.30)	0.20(0.08, 0.32)	0.19(0.01,0.30)	0.332
ΔTriglycerides, mmol/L	− 0.25 (− 0.67, 0.16)	− 0.41 (− 0.90, − 0.02)	− 0.20 (− 0.56, 0.19)	0.037

Values are expressed as the median (interquartile range) for continuous variables with abnormal distribution and described as the mean ± standard deviation with normal distribution, or frequency (percentage) for categorical variables in the table.

*ACEI* angiotensin-converting enzyme inhibitor, *ARB* angiotensin receptor blocker, *CCB* calcium channel blocker, *DM* diabetes mellitus, *eGFR* estimated glomerular filtration rate, *HDL-C* high-density lipoprotein cholesterol, *LDL-C* low-density lipoprotein cholesterol.

**Table 2:** Patient demographics in phase II analysis.

	Total (N = 114)	DM group (n = 42)	Non-DM group (n = 72)	*p* value
Age, years	70.00 [63.00, 78.00]	70.50 [66.00, 77.00]	70.00 [60.25, 78.00]	0.993
Men n (%)	89 (78.1)	31(73.8)	58(80.5)	0.401
Body mass index	25.30 [23.10, 27.19]	25.09 [23.24, 27.20]	25.39 [22.94, 26.98]	0.953
Hypertension, n (%)	80 (70.2)	30 (71.4)	50 (69.4)	0.823
Dyslipidemia, n (%)	70 (61.4)	24 (57.1)	46 (63.8)	0.475
Smoking, n (%)	38 (33.3)	10(23.8)	28 (38.8)	0.099
Baseline medical therapy				
Statin, n (%)	114 (100.0)	42 (100.0)	112 (100.0)	NA
Ezetimibe, n (%)	35 (30.7)	12 (28.6)	23 (31.9)	0.706
Antiplatelet therapy, n (%)	114 (100.0)	42 (100.0)	112 (100.0)	NA
Clopidogrel, n (%)	78 (68.4%)	21(50.0%)	57(79.2%)	0.001
Ticagrelor, n (%)	36 (31.6)	21(50.0)	15(20.8)	0.001
ACEI/ARB, n (%)	66 (57.9)	25(59.5)	41 (56.9)	0.788
CCB, n (%)	31 (27.2)	10 (23.8)	21 (29.2)	0.535
Beta-blocker, n (%)	64 (56.1)	27(64.3)	37 (51.4)	0.181
Baseline lipid profile				
Total cholesterol, mmol/L	3.56 ± 0.77	3.64 ± 0.82	3.51 ± 0.74	0.403
LDL-C, mmol/L	1.99 ± 0.66	2.09 ± 0.72	1.92 ± 0.61	0.202
HDL-C, mmol/L	1.03 ± 0.24	1.03 ± 0.20	1.03 ± 0.26	0.913
Non-HDL-C, mmol/L	2.53 ± 0.73	2.61 ± 0.78	2.92 ± 0.98	0.130
Triglycerides, mmol/L	1.62 ± 0.93	1.56 ± 0.77	1.65 ± 1.02	0.615
Glucose, mmol/L	6.02 ± 2.34	7.74 ± 3.00	5.03 ± 0.90	< 0.001
Serum creatinine	–	74.41 ± 17.34	75.86 ± 18.59	0.683
eGFR, ml	–	94.77 ± 27.62	94.70 ± 23.59	0.989
60 ml ≤ eGFR < 90 ml, n (%)	–	17 (40.5)	26 (36.1)	0.643
Location of nCSA				
LAD, n (%)	79 (69.3)	34 (80.9%)	45 (62.5%)	0.111
LCx, n (%)	11 (9.6)	2 (4.8%)	9 (12.5%)	
RCA, n (%)	24 (21.1)	6 (14.3%)	18 (25.0%)	
1-year follow up lipid profile				
Total cholesterol, mmol/L	3.59 ± 0.91	3.79 ± 1.07	3.47 ± 0.59	0.069
LDL-C, mmol/L	1.89 ± 0.71	2.05 ± 0.86	1.80 ± 0.59	0.077
HDL-C, mmol/L	1.19 ± 0.25	1.18 ± 0.21	1.19 ± 0.27	0.913
LDL-C < 1.4 mmol/L, n (%)	30(26.4)	10(23.8)	20(27.8)	0.428
Non-HDL-C, mmol/L	2.41 ± 0.88	2.65 ± 1.04	2.26 ± 0.74	0.052
Triglycerides, mmol/L	1.31 ± 0.56	1.28 ± 0.52	1.33 ± 0.58	0.615
Glucose, mmol/L	6.24 ± 1.78	7.56 ± 2.13	5.45 ± 0.85	< 0.001
Change in lipid profile between index and 1-year follow-up				
ΔTotal cholesterol, mmol/L	0.00 (− 0.44, 0.59)	0.26 (− 0.38, 0.84)	− 0.10 (− 0.44, 0.17)	0.069
ΔLDL-C, mmol/L	− 0.08 (− 0.41, 0.26)	0.00 (− 0.40, 0.52)	− 0.16 (− 0.44, 0.17)	0.259
ΔLDL-C%	− 0.05 (− 22.79,14.39)	0.00 (− 25.66,21.58)	− 0.09 (− 22.08,11.27)	0.414
Δnon-HDL-C, mmol/L	− 0.11 (− 0.53,0.37)	0.05 (− 0.54,0.68)	− 0.18 (− 0.54,0.16)	0.083
ΔHDL-C, mmol/L	0.15 (0.04, 0.27)	0.17 (0.02, 0.25)	0.15 (0.05, 0.30)	0.718
ΔTriglycerides, mmol/L	− 0.17 (− 0.54, 0.17)	− 0.21 (− 0.59, 0.13)	− 0.14 (− 0.52, 0.25)	0.455

Values are expressed as the median (interquartile range) for continuous variables with abnormal distribution and described as the mean ± standard deviation with normal distribution, or frequency (percentage) for categorical variables in the table.

*ACEI* angiotensin-converting enzyme inhibitor, *ARB* angiotensin receptor blocker, *CCB* calcium channel blocker, *DM* diabetes mellitus, *eGFR* estimated glomerular filtration rate, *HDL-C* high-density lipoprotein cholesterol, *LDL-C* low-density lipoprotein cholesterol.

### OCT measurement and OFR analysis in phase II analysis

All the OCT measurement data and OFR analysis for nCSA are summarized in Table 3. Baseline morphologies such as TAVn (119.24 ± 40.68 mm^3^ vs. 115.81 ± 39.37 mm^3^, *p* = 0.659), PAV (44.51 ± 8.00% vs. 44.79 ± 8.80%, *p* = 0.866) and composition of nCSAs such as lipid TAVn, fibrous TAVn/PAV, fibrous TAVn/PAV, macrophage TAVn/PAV and TFCT were comparable between the DM and non-DM groups, except lipid PAV (22.95 (17.53, 28.43)% vs. 16.15 (10.55, 25.50)%, *p* = 0.026) was higher in the DM group than in the non-DM group at baseline.

**Table 3:** nCSA changes measured by OFR for patient with or without DM in phase II analysis.

Parameter	DM group (n = 42)	Non-DM group (n = 72)	*p* value
TAVn, mm^3^			
Baseline, mm^3^	119.24 ± 40.68	115.81 ± 39.37	0.659
1-year follow-up, mm^3^	126.16 ± 45.32**	115.96 ± 40.97	0.231
ΔTAVn, mm^3^	7.41 [− 2.82, 11.85]	− 1.12 [− 10.67, 9.15]	0.009
PR ratio based on ΔTAVn, %	13(31.0)	37(51.4)	0.034
PAV, %			
Baseline, %	44.51 ± 8.00	44.79 ± 8.80	0.866
1-year follow-up, %	46.30 ± 7.60*	44.22 ± 8.87	0.214
ΔPAV, %	1.55 [− 0.80, 3.63]	− 1.15 [− 3.00, 2.25]	0.008
PR ratio based on ΔPAV, %	16(38.1)	42(58.3)	0.037
Lipid TAVn, mm^3^			
Baseline, mm^3^	23.55 [16.19, 40.27]	18.41 [8.32, 30.23]	0.053
1-year follow-up, mm^3^	24.26 [15.05, 40.12]	16.03 [6.98, 26.54]	0.004
Lipid ΔTAVn, mm^3^	0.32 [− 6.74, 10.03]	− 2.63 [− 7.99, 4.59]	0.140
Lipid PAV, %			
Baseline, %	22.95 [17.53, 28.43]	16.15 [10.55, 25.50]	0.026
1-year follow-up, %	21.95 [15, 48, 26.83]	14.70 [8.28, 24.50]	0.002
Lipid ΔPAV, %	0.70 [− 5.88, 3.45]	− 1.85 [− 7.08, 3.13]	0.214
Fibrous TAVn, mm^3^			
Baseline, mm^3^	72.47 [54.70, 92.21]	70.73 [58.66, .92.03]	0.724
1-year follow-up, mm^3^	76.83 [58.70, 105.60]*	74.72 [61.14, 95.69]***	0.664
Fibrous ΔTAVn, mm^3^	4.30 [− 4.14, 10.69]	3.99 [− 2.81, 8.94]	0.916
Fibrous PAV,%			
Baseline, %	64.95 [55.75, 71.35]	69.10 [57.35, 76.45]	0.063
1-year follow-up, %	66.70 [57.68, 73.83]	71.70 [62.40, 79.75]***	0.006
Fibrous ΔPAV,%	0.45 [− 5.20, 7.98]	2.70 [− 1.95, 9.08]	0.178
Calcium TAVn, mm^3^			
Baseline, mm^3^	0.45 [0.08, 2.65]	0.54 [0.01, 2.49]	0.871
1-year follow-up, mm^3^	1.02 [0.05, 6.73]**	0.55 [0.08, 3.29]	0.395
Calcium ΔTAVn, mm^3^	0.26 [− 0.08, 3.22]	0.00 [− 0.14, 0.90]	0.098
Calcium PAV, %			
Baseline, %	0.30 [0.10, 2.40]	0.55 [0.00, 2.45]	0.880
1-year follow-up, %	0.75 [0.01, 5.10]*	0.60 [0.10, 3.38]*	0.480
Calcium ΔPAV, %	0.10 [− 0.20, 2.23]	0.00 [− 0.10, 0.85]	0.451
Macrophage TAVn, mm^3^			
Baseline, mm^3^	0.42 [0.18, 0.78]	0.41 [0.11, 0.96]	0.904
1-year follow-up, mm^3^	0.56 [0.14, 1.17]	0.32 [0.07, 0.61]	0.040
Macrophage ΔTAVn, mm^3^	0.04 [− 0.19, 0.55]	− 0.06 [− 0.41, 0.79]	0.057
Macrophage PAV, %			
Baseline, %	0.50 [0.10, 0.73]	0.35 [0.10, 0.80]	0.991
1-year follow-up, %	0.35 [0.20, 0.90]	0.25 [0.10, 0.67]	0.085
Macrophage ΔPAV, %	− 0.05 [− 0.20, 0.30]	− 0.05 [− 0.38, 0.10]	0.095
TFCT, µm			
Baseline, µm	100.00 [58.50, 129.50]	111.00 [78.50, 134.00]	0.481
1-year follow-up, µm	115.50 [69.25, 175.00]	120.00 [86.00, 167.50]*	0.640
ΔTFCT, µm	16.50 [− 35.50, 82.75]	15.50 [− 15.50, 51.25]	0.758
MLA, mm^2^			
Baseline, mm^2^	5.09 ± 2.36	5.37 ± 3.33	0.607
1-year follow-up, mm^2^	4.62 ± 2.23**	5.23 ± 3.19	0.270
ΔMLA, mm^2^	− 0.38 [− 0.75, 0.11]	− 0.05 [− 0.46, 0.42]	0.020

Values are expressed as the median (interquartile range) for continuous variables with abnormal distribution and described as the mean ± standard deviation with normal distribution, or frequency (percentage) for categorical variables in the table.

*DM* diabetes mellitus, *MLA* minimal luminal area, *nCSA* non-culprit subclinical atherosclerosis, *OFR* optical flow ratio, *PAV* percent atheroma volume, *TAVn* normalized total atheroma volume, *TFCT* thinnest fibrous cap thickness.

***p < 0.001; **p < 0.01; *p < 0.05 these values were compared between baseline and 1-year follow-up.

Even though LDL-C was reduced by a similar amount in both DM and non-DM patients in the second-stage analysis (0.00 (− 0.40, 0.52) mmol/L vs. − 0.16 (− 0.44, 0.17) mmol/L, *p* = 0.259), reaching similar final levels of LDL-C at the 1-year follow-up (2.05 ± 0.86 mmol/L vs. 1.80 ± 0.59 mmol/L, *p* = 0.077), patients with DM showed more PP of nCSA morphologies at the 1-year follow-up, which, expressed as ΔTAVn, was higher in the DM group than in the non-DM group from baseline to the 1-year follow-up (7.41 (− 2.82, 11.85) mm^3^ vs. − 1.12 (− 10.67, 9.15) mm^3^, *p* = 0.009). It was further analysed that plaque progression caused the TAVn to change significantly in the DM group (126.16 ± 45.32 mm^3^ vs. 119.24 ± 40.68 mm^3^, *p* = 0.004) rather than non-significantly in the non-DM group (115.96 ± 40.97 mm^3^ vs. 115.81 ± 39.37 mm^3^, *p* = 0.938) at the 1-year follow-up compared to baseline data. Moreover, ΔPAV was greater in the DM group than in the non-DM group from baseline to the 1-year follow-up (1.55 (− 0.80, 3.63)% vs. − 1.15 (− 3.00, 2.25)%, *p* = 0.008), and further analysis of the internal reason showed that PAV increased significantly in the DM group (46.30 ± 7.60% vs. 44.51 ± 8.00%, *p* = 0.014) rather than non-significantly in the non-DM group (44.22 ± 8.87% vs. 44.79 ± 8.80%, *p* = 0.375) at the 1-year follow-up compared to baseline data. Another interesting finding in our study was that the MLA in the analysed segment decreased significantly in the DM group (5.09 ± 2.36 mm^2^ vs. 4.62 ± 2.23 mm^2^, *p* = 0.001) and non-significantly in the non-DM group (5.37 ± 3.33 mm^2^ vs. 5.23 ± 3.19 mm^2^, *p* = 0.766) from baseline to the 1-year follow-up, and the ΔMLA decreased significantly in the DM group compared to that in the non-DM group (− 0.38 (− 0.75, 0.11) mm^2^ vs. − 0.05 (− 0.46, 0.42) mm^2^, *p* = 0.020), which indicated significant late lumen loss in the DM group.

Further analysis of changes in nCSA composition revealed that even lipid TAVn increased in the DM group non-significantly (23.55 (16.19, 40.27) mm^3^ vs. 24.26 (15.05, 40.12) mm^3^, *p* = 0.280) rather than decreased in the non-DM group non-significantly (18.41 (8.32, 30.23) mm^3^ vs. 16.03 (6.98, 26.54) mm^3^, *p* = 0.128), even though lipid ΔTAVn was not significantly different between DM and non-DM groups from baseline to 1-year follow-up (0.32 (− 6.74,10.03) mm^3^ vs. − 2.63 (− 7.99, 4.59) mm^3^, *p* = 0.140), lipid TAVn was higher (*p* = 0.004) in the DM group than in the non-DM group, accompanied by an increase in lipid PAV (21.95 (1548, 26.83)% vs. 14.70 (8.28, 24.50)%, *p* = 0.002) at the 1-year follow-up. Other nCSA composition changes, such as fibrous ΔTAVn/ΔPAV, calcium ΔTAVn/ΔPAV, macrophage ΔTAVn/ΔPAV, and ΔTFCT, showed no significant changes between the DM and non-DM groups, except that the fibrous PAV was significantly higher (70.40 (62.40, 79.75)% vs. 66.70 (57.68, 73.83)%, *p* = 0.006) and macrophage TAVn was significantly lower (0.32 (0.07, 0.61) mm^3^ vs. 0.56 (0.14, 1.17) mm^3^, *p* = 0.040) in the non-DM group at the 1-year follow-up.

**Figure 2:**
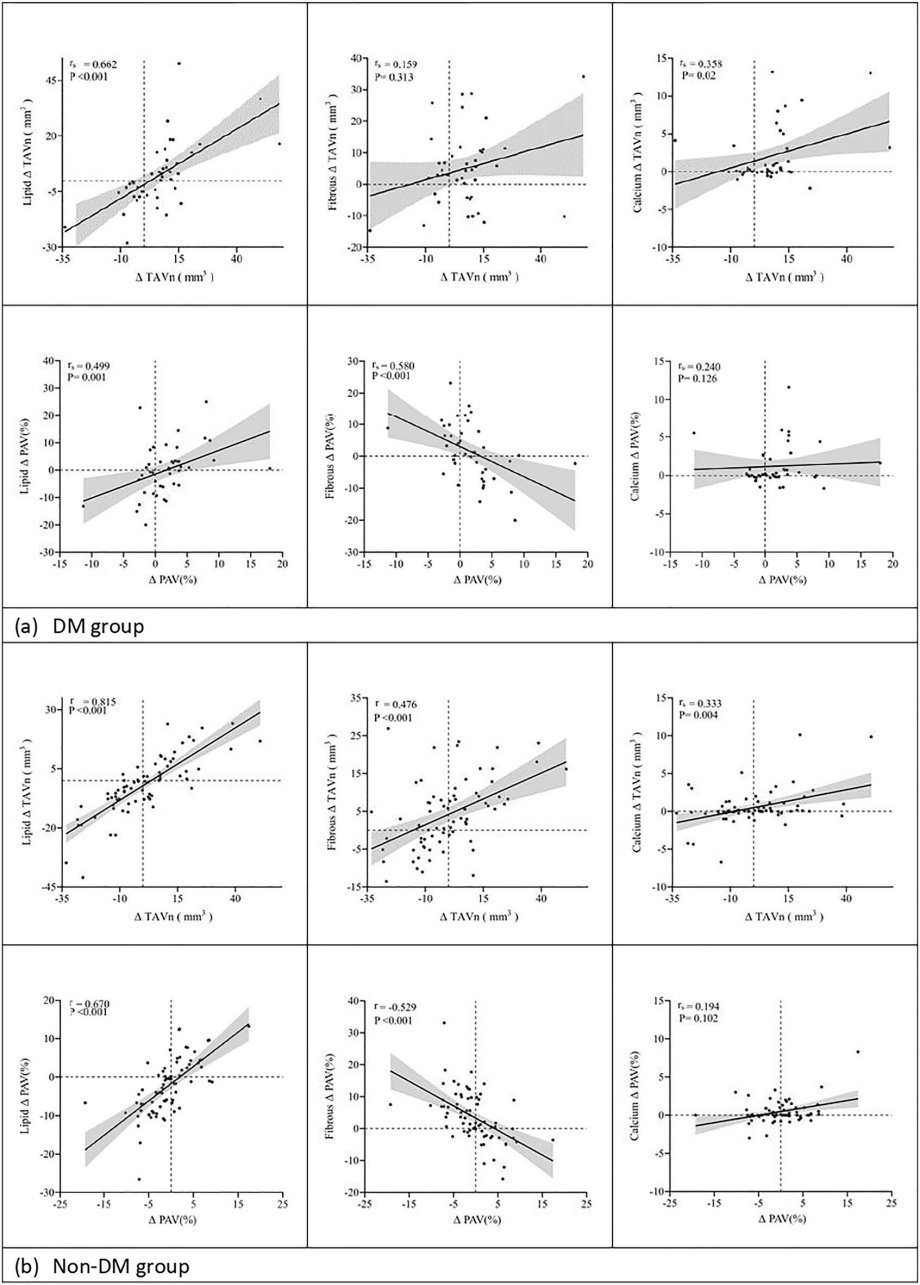
Correlation between changes of nCSA and each component in the DM group (**a**) and non-DM group (**b**).

**Figure 3:**
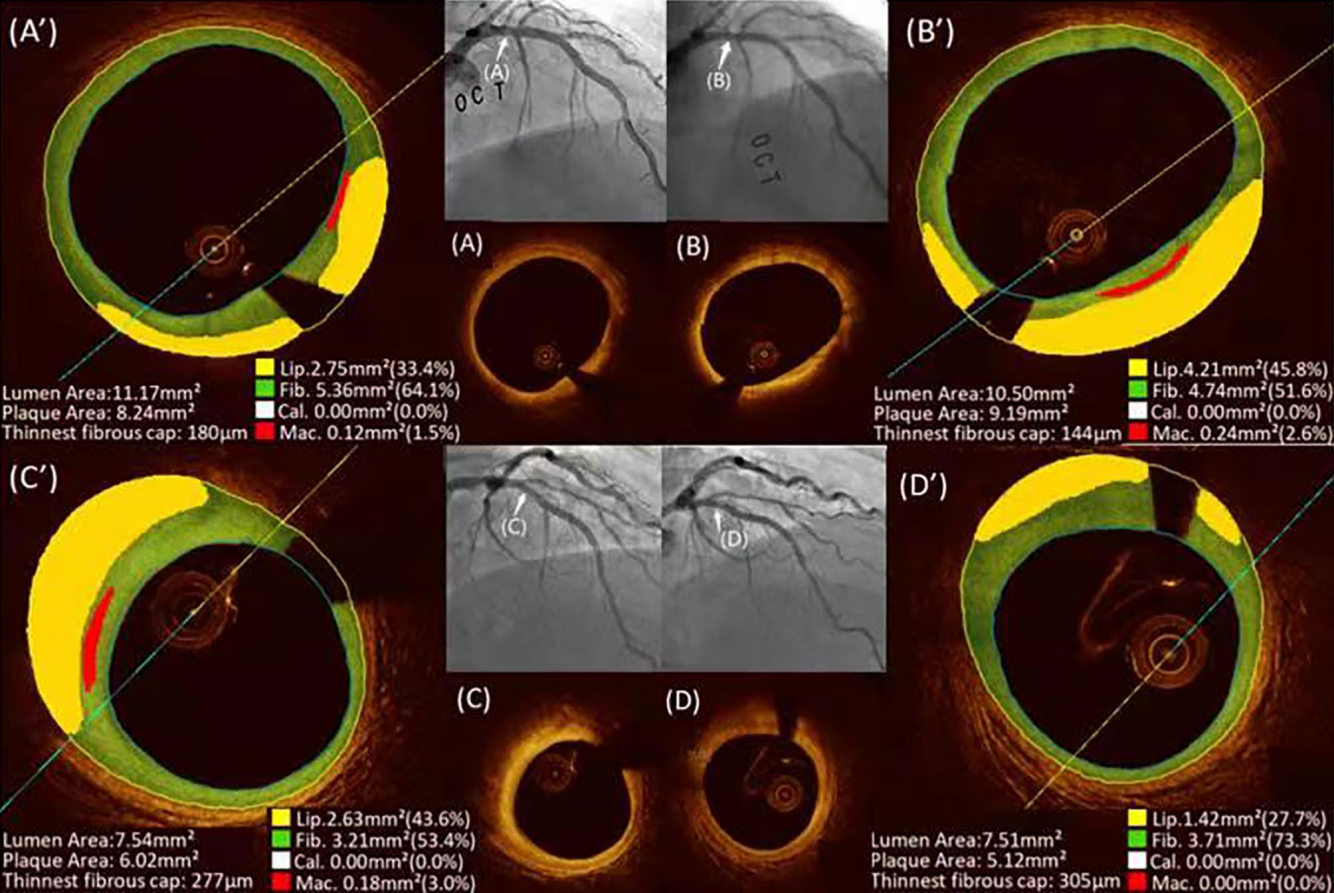
Typical cases of cross-sectional OCT imaging in nCSA of DM (**A**, **B**) and non-DM (**C**, **D**) at baseline and 1-year follow-up. Representative cross-sectional OCT image in DM group demonstrating increase in the lipid component from baseline (original OCT image (**A**) and OCT image by OFR analysis (**A**’) at baseline showed lipid plaque area 2.75 mm^2^ and lipid plaque burden 33.4%) to 1-year follow-up (OCT image (**b**) and OCT image by OFR analysis (**B**’) showed lipid plaque area 4.21 mm^2^ and lipid plaque burden 45.8%). Representative cross-sectional OCT image in DM group demonstrating reduction in the lipid component from baseline (original OCT image (**c**) and OCT image by OFR analysis (**C**’) at baseline showed lipid plaque area 2.63 mm^2^ and lipid plaque burden 43.6%) to 1-year follow-up (OCT image (**d**) and OCT image by OFR analysis (**D**’) showed lipid plaque area 1.42 mm^2^ and lipid plaque burden 27.7%). *DM* diabetes mellitus, *OCT* optical coherence tomography, *OFR* optical flow ratio.

In the DM group, ΔTAVn was significantly positively correlated only with lipid ΔTAVn (r = 0.662, *p* < 0.001) and calcium ΔTAVn (r = 0.358, *p* = 0.02), but there was no significant correlation with fibrous ΔTAVn (r = 0.159, *p* = 0.313); furthermore, ΔPAV was found to be significantly positively correlated with lipid ΔPAV (r = 0.499, *p* = 0.001) rather than negatively correlated with fibrous ΔPAV (r = − 0.58, *p* < 0.001), and there was no significant correlation with calcium ΔPAV (r = 0.24, *p* = 0.126) (Fig. 2a). In the non-DM group, ΔTAVn was significantly positively correlated with lipid ΔTAVn (r = 0.815, *p* < 0.001), fibrous ΔTAVn (r = 0.476, *p* < 0.001), and calcium ΔTAVn (r = 0.333, *p* = 0.004); furthermore, ΔPAV was found to be significantly positively correlated with lipid ΔPAV (r = 0.670, *p* < 0.001) rather than negatively correlated with fibrous ΔPAV (r = − 0.529, *p* < 0.001), and there was no significant correlation with calcium ΔPAV (r = 0.194, *p* = 0.102) (Fig. 2b). There were two typical examples of natural morphologies and composition in nCSA changes in DM patients and non-DM patients at baseline and 1-year follow-up (Fig. 3), which showed that lipid area increased from 2.75 to 4.21 mm^2^ combined with plaque area increased from 8.24 to 9.19 mm^2^ and luminal cross sectional area decreased from 11.17 to 10.50 mm^2^ in DM group, otherwise, lipid area decreased from 2.63 to 1.42 mm^2^ combined with plaque area decreased from 6.2 to 5.12 mm^2^ and no changes in luminal cross sectional area from 7.54 to 7.51 mm^2^.

### Exploratory analysis of the clinical factors leading to nCSA-related MACEs at the 3-year follow-up

Univariate correlation analysis showed that DM, sex, age, LDL-C level at the 1-year follow-up, ΔLDL-C% and the use of ezetimibe had a potential correlation with the PP of nCSA; these factors were then transferred to the multiple regression analysis to compute the standardized coefficients. Finally, DM (OR = 2.731, 95% CI 1.160–6.428, *p* = 0.021) and the use of ezetimibe (OR = 2.916, 95% CI 1.187–7.162, *p* = 0.020) were correlated with the PP of nCSA (Table 4).

**Table 4:** Multivariate logistic regression analysis of factors predicting plaque progression of nCSA.

Variable	β	S0.E	Wald	*P*	OR	95% CI	
						Low	High
Diabetes	1.005	0.437	5.290	0.021	2.731	1.160	6.428
Gender	0.783	0.502	2.433	0.119	2.188	0.818	5.853
Age	0.013	0.020	0.382	0.537	1.013	0.973	1.054
Follow-up LDL-C (mmol/L)	-0.084	0.343	0.060	0.807	0.919	0.470	1.800
ΔLDL-C%	0.519	0.860	0.364	0.546	1.681	0.311	9.071
Ezetimibe	1.070	0.458	5.450	0.020	2.916	1.187	7.162
Constant	− 1.729	1.648	1.101	0.294	0.178		

*CI* confidence interval, *LDL-C* low-density lipoprotein cholesterol, *OR* odds ratio, *PP* plaque progression.

**Figure 4:**
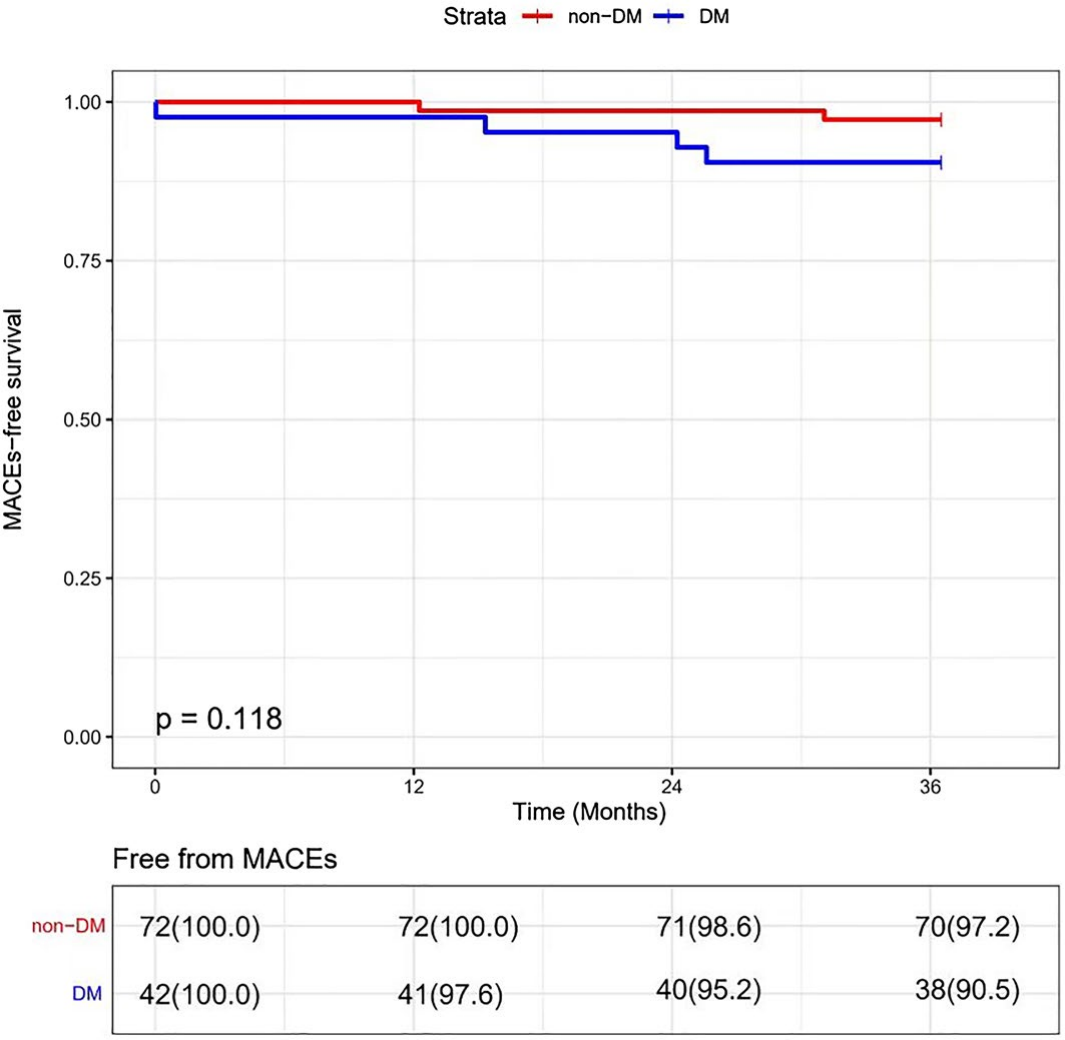
MACEs-free related to nCSA survival during the 3-year follow-up between DM and non-DM in phase II.

**Figure 5:**
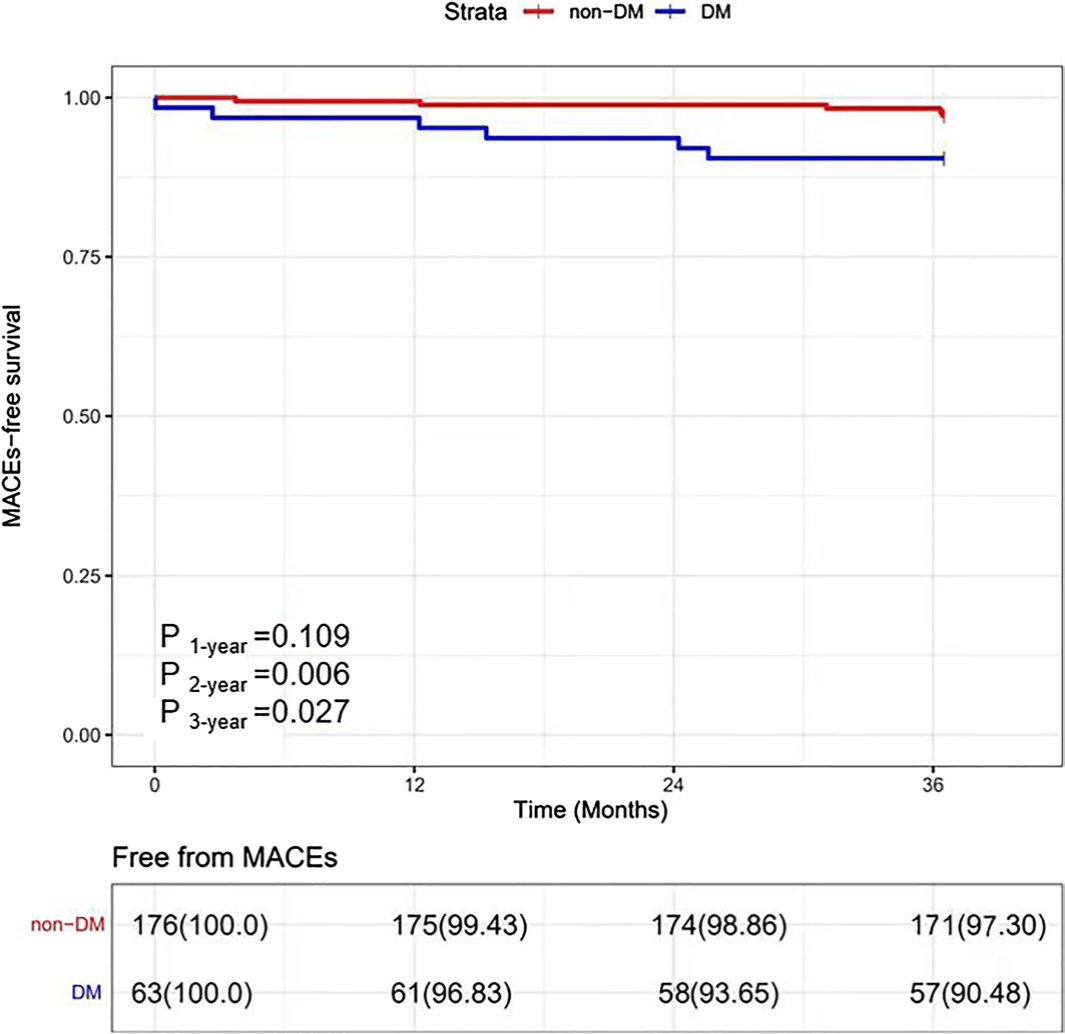
MACEs-free related to nCSA survival during the 3-year follow-up between DM and non-DM in phase I.

The 3-year clinical follow-up for the patients with 1–2 nCSA results showed an increased tendency of the incidence of MACEs related to nCSA in the DM group than those in the non-DM group, but there was no significant difference (9.5% vs. 2.8%, *p* = 0.118) by Kaplan–Meier analysis, even with a commensurable LDL-C reduction at the 1-year follow-up (1.80 ± 0.59 mmol/L vs. 2.05 ± 0.86 mmol/L, *p* = 0.077) for the patients in the phase II (Fig. 4 and Table 2). When we expanded the sample size to phase I analysis, there was a significant increase of the incidence of 3-year's MACEs related to nCSA in the DM group than those in the non-DM group (9.5% vs. 1.7%, *p* = 0.027), with a similar LDL-C reduction in both groups at the 1-year follow-up (1.94 ± 0.69 mmol/L vs. 1.95 ± 0.74 mmol/L, *p* = 0.925) and comparable baseline morphologies and composition of nCSA in both groups (such as TAVn, PAV, lipid component, calcium component, macrophage component, et al.) for the total population in the phase I analysis (Fig. 5, Tables 1, 5).

**Table 5:** nCSA baseline characteristics measured by OFR for patients with or without DM in phase I analysis.

Baseline parameter	DM group (n = 63)	Non-DM group (n = 176)	*p* value
TAVn, mm^3^	117.54 ± 40.56	115.96 ± 40.42	0.826
PAV, %	44.88 ± 8.49	44.34 ± 8.56	0.726
Lipid TAVn, mm^3^	23.21 [16.04,38.17]	20.20 [9.05,32.14]	0.231
Lipid PAV, %	21.61 [15.80,28.20]	18.90 [11.35,26.85]	0.149
Fibrous TAVn, mm^3^	71.01 [54.75, 90.62]	71.85 [56.37,91.50]	0.733
Fibrous PAV, %	63.55 ± 12.16	66.24 ± 13.10	0.238
Calcium TAVn, mm^3^	0.53 [0.08, 2.86]	0.38 [0.02, 2.56]	0.648
Calcium PAV, %	0.44[0.10, 2.48]	0.40[0.00, 2.35]	0.638
Macrophage TAVn, mm^3^	0.41[0.17, 0.75]	0.42[0.20, 1.02]	0.674
Macrophage PAV, %	0.45 [0.13, 0.90]	0.40[0.10, 0.80]	0.716
TFCT, µm	100.00 [58.50, 129.50]	111.00 [78.50, 134.00]	0.481
MLA, mm^2^	5.09 ± 2.36	5.37 ± 3.33	0.607

Values are expressed as the median (interquartile range) for continuous variables with abnormal distribution and described as the mean ± standard deviation with normal distribution, or frequency (percentage) for categorical variables in the table.

*DM* diabetes mellitus, *MLA* minimal luminal area, *nCSA* non-culprit subclinical atherosclerosis, *OFR* optical flow ratio, *PAV* percent atheroma volume, *TAVn* normalized total atheroma volume, *TFCT* thinnest fibrous cap thickness.

## Discussion

The main findings of this study are as follows:

1. TAVn morphologies of nCSA in DM patients are more likely to progress, which is mainly due to the increase in lipid components, than those in non-DM patients from baseline to the 1-year follow up despite LLT treatment with statins and a similar LDL-C level reduction.
2. The incidence of MACEs related to nCSA was higher in patients with DM than in patients without DM at the 2- and 3-year follow-up.

With the development of IVI in the study of coronary plaque, nCSA has received increasing attention because the MACEs caused by nCSA are comparable to culprit lesions at the 3-year follow-up^43^. Therefore, stabilizing and regressing nCSA to reduce the incidence of corresponding MACEs is our goal, especially for patients with ACS post PCI for culprit lesions^9,26,44,45^. Although it has been well known for a long time that DM is a coronary artery disease equivalent in clinical practice^9,40,46^, which easily causes not only coronary PP but also plaque instability^47,48^, early initiation of LLT with statins is targeted at such patients^39,40,46,49^. Many previous studies have demonstrated that intensive LLT leads to a significant PR measured by TAVn/PAV based on IVUS or OCT data for patients with intermediate or moderator stenotic lesions^50–53^; however, even when LLT based on statins has been tried in DM patients, it is not as effective as in non-DM patients^14,54–56^, and coronary PP could also be found in patients with DM despite obtaining very low levels of LDL-C^48^. There is a consensus on the impact of DM on coronary heart disease in clinical practice, and LDL-C level control based on LLT in the real world is still not satisfactory, which is far from the standard recommended by the guidelines^42,49,57^. The main cause of recurrent MACEs in ACS patients is not only culprit lesions; approximately 50% of the causes are from the progression of nonculprit lesions^43^, and more aggressive LLT (including statins, ezetimibe, and PCSK9i) is conducive to the regression of nonculprit lesions^58,59^. Patients with PR have a significantly lower 1–3-year incidence of cardiac events^18,60–62^. Therefore, the outcome of nCSA in ACS patients after PCI for culprit lesions is a key issue that our clinicians should focus on.

The development in data analytics and digitized medical imaging has enabled the clinical application of AI to improve the acute judgement of the characteristics of coronary lesions and optimization of the PCI procedure, which can improve patient outcomes^27,28,63^. OFR is an AI software developed based on OCT data analysis in recent years that can automatically identify the morphology and composition of coronary plaque^18,27,28^. In this observational study, we used this software to retrospectively analyse the OCT data of our previous RCT studies based on the concept of AI. By comparing baseline and 1-year follow-up OCT data of nCSA in ACS patients, the results showed that the ΔTAVn/ΔPAV of nCSA in DM patients was significantly higher than that in non-DM patients, even when the clinical baseline data were matched, and the level of lipid decline was comparable. The main reason for this difference, as shown in Table 3, is that TAVn/PAV increased significantly from baseline to the 1-year follow-up in the DM group, whereas it did not change significantly in the non-DM group. Similar results were obtained in previous studies based on IVUS findings^14,48,64^. Compared with non-DM patients, both plaque burden and plaque risk profiles were found to accelerate progression in DM patients, and the increase was more pronounced than in non-DM patients over a longer period of follow-up, which would increase MACEs slowly. Another important reason for this phenomenon is that the LDL-C level compliance rate (< 1.4 mmol/L) at the 1-year follow-up after LLT based on statins according to several guidelines is very low^39,40,42^. As shown in our study, with continued LLT based on statins, less than 30% of patients had an LDL-C level < 1.4 mmol/L at the 1-year follow-up. Similar results were shown in previous studies, which showed similar plaque outcomes detected by IVUS or OCT^14–16^. Of course, more intensive LLT combined with PCSK9i leads not only to significant reductions in LDL-C effectively compared with non-DM patients but also to regression in plaque volume in non-DM patients, but there is still a lack of evidence that patients with DM have the same effect as those without DM^58,59,65,66^.

To study the intrinsic factors of nCSA change differences between DM and non-DM patients, we further analysed the plaque composition using OFR software by comparing each component value of lipid/fibrous/calcium/macrophage from baseline to the 1-year follow-up in the same segment after manual multiaspect coregistration. The lipid component of lipid TAVn increased from the baseline non-significantly in the DM group; meanwhile, there was a non-significant reduction in the non-DM group at 1-year follow-up. Integrated final result showed that lipid TAVn was significantly higher in the DM group than in the non-DM group (*p* = 0.004). This notion was echoed in a previous integrated backscatter IVUS-based study^15^. Regarding potential clinical significance, it is reasonable to suggest that routine LLT in DM patients cannot prevent the increase in lipid components in nCSA when compared with non-DM patients. Further univariate correlation analysis showed that the increase in ΔTAVn in the DM group was mainly positively related to the increase in lipid and calcium components but not related to changes in fibrous components. However, the relative increase in the nCSA volume of ΔPAV in the DM group was mainly caused by an increase in the lipid component (which was supported by a previous study)^16^, while there was a reduction in the fibrous component and no significant change in the calcium component. The situation was slightly different in the non-DM group; the increase in ΔTAVn was positively related to all lipid, fibrous and calcium components, but the relative increase in the nCSA volume of ΔPAV was similar to that of the DM group, which meant that the changes in the lipid component were the greatest. Another potential factor may be that macrophage ΔTAVn was significantly higher in the DM group than in the non-DM group at the 1-year follow-up, although baseline levels were comparable in the two groups. This idea has been confirmed by previous studies, which showed that inflammation is one of the main factors leading to PP and was higher in the DM group than in the non-DM group^9,12,26,67^. Although the use of ezetimibe seemed to be a risk factor for predicting the PP of nCSA at the 1-year follow-up, we think that the specific plan to administer LLT with statins was decided by the doctor for each patient, but the concern is that doctors prescribe the combination of ezetimibe and statins to patients when the efficacy of statins alone is unsatisfactory. The real conclusion should be that ezetimibe is used in patients with unsatisfactory lipid profile control rather than for the PP of nCSA; in other words, if such patients do not use ezetimibe, nCSA will make progress further. Therefore, the effect of ezetimibe on the outcome of nCSA needs to be interpreted with caution, and it cannot be concluded simply that ezetimibe is related to nCSA progression from multivariate logistic regression analysis, what is more important is the effect and duration of lipid lowering effect^52^.

Another important finding in this study (in phase I analysis) is that the incidence of MACEs at the 3-year follow-up related to nCSA was higher in the DM group than in the non-DM group, even though this difference was not found at the 1-year follow-up. Based on a previous analysis of OCT data (OFR measurement), depending on our phase II analysis result, which showed that ΔMLA at the analysis segment of nCSA was significantly increased at the 1-year follow-up, the MLA value at the 1-year follow-up was still acceptable and did not cause ischaemia.

We hypothesized that the main reason was that conventional LLT did not effectively prevent PP of nCSA, and the unchanged long-term LLT regimen might delay the deterioration of nCSA, resulting in a significant increase in corresponding MACEs at the 3-year follow-up. This finding suggests that for DM patients with ACS, if the culprit lesion is treated by PCI and nCSA is only treated with conventional LLT, even if there are no MACEs in 1 year, the incidence of MACEs will increase significantly after 2–3 years of follow-up, which is closely related to the PP of nCSA at the 1-year IVI follow-up. Because the lipid component is the main factor of PP, it is possible that more intensive LLT may have a better effect^68^, but this reduction in nCSA-related MACEs for patients with DM needs to be confirmed by further randomized controlled studies.

Additionally, the implication of this study is that for patients with DM combined with coronary artery disease, more intensive LLT and anti-inflammation therapy for PR in nCSA is needed. If PP in nCSA is found at the 1-year follow-up by IVI, it indicates that LLT was not fully beneficial in the early stage, and further intensive treatment is needed; otherwise, the incidence of nCSA-related MACEs will increase significantly at the 3-year follow-up.

## Conclusions

Morphological changes in the nCSA by TAVn measurement in DM patients are more likely to progress than those in non-DM patients from baseline to the 1-year follow-up despite LLT treatment with statins and a similar LDL-C level reduction. The main composition of the lipid component of the nCSA is more likely to increase in patients with DM than in non-DM patients at the 1-year follow-up. The incidence of MACEs related to nCSA was higher in patients with DM than in patients without DM at the 3-year follow-up.

## Limitations

The number of cases in this study was relatively small, with a lack of follow-up results on the effect of blood glucose control in the DM group, and inflammatory factors were also lacking. This study is a subgroup analysis of our previous randomized controlled study focusing on subclinical stent thrombosis. The clinical protocol for LLT after PCI is not strict, which leads to a low proportion (< 30%) of the final LDL-C level in this study meeting the current guideline recommendation. The number of cases detected by OCT for nCSA at baseline and the 1-year follow-up is a fraction of the overall population in the study and theoretically cannot fully reflect the changes and outcomes of nonculprit lesions in all patients (DM and non-DM). Although the OFR software is a novel AI framework for not only the measurement of local coronary functional indexes but also automatic plaque composition detection, which is similar to histopathological analysis confirmed by previous studies, the clinical application of this software is rapid and simple, and its clinical value needs to be confirmed in more studies.

## Data Availability

Data supporting the findings of this study are available from Nanjing Hospital Affiliated to Nanjing Medical University, but the availability of these data is limited, and they were used under license for the current study and therefore are not publicly available. However, data are available from the authors upon reasonable request and with permission from the Affiliated Nanjing Hospital of Nanjing Medical University.

## Code availability

Code is available.
